# Synergistic effect of biological and advanced oxidation process treatment in the biodegradation of Remazol yellow RR dye

**DOI:** 10.1038/s41598-020-77376-5

**Published:** 2020-11-19

**Authors:** Muruganandham Thanavel, Paul Olusegun Bankole, Ramu Selvam, Sanjay Prabhu Govindwar, Senthil Kumar Sadasivam

**Affiliations:** 1 Post Graduate and Research Department of Biotechnology, National College (Autonomous), Dindigul Road, Tiruchirappalli, Tamil Nadu 620 001 India; 2 Department of Pure and Applied Botany, College of Biosciences, Federal University of Agriculture, P.M.B. 2240, Abeokuta, Ogun State Nigeria; 3 Department of Earth Resources and Environmental Engineering, Hanyang University, Seoul, 04763 South Korea; 4 Post Graduate and Research Department of Botany, National College (Autonomous), Dindigul Road, Tiruchirappalli, Tamil Nadu 620 001 India

**Keywords:** Biochemistry, Biotechnology, Environmental sciences

## Abstract

The current study investigated the efficiency of synergistic biological and Advanced Oxidation Process (AOPs) treatment (B-AOPs) using *Aeromonas hydrophila* SK16 and AOPs-H_2_O_2_ in the removal of Remazol Yellow RR dye. Singly, *A. hydrophila* and AOPs showed 90 and 63.07% decolourization of Remazol Yellow RR dye (100 mg L^−1^) at pH 6 and ambient temperature within 9 h respectively. However, the synergistic B-AOPs treatments showed maximum decolorization of Remazol Yellow RR dye within 4 h_._ Furthermore, the synergistic treatment significantly reduced BOD and COD of the textile wastewater by 84.88 and 82.76% respectively. Increased levels in laccase, tyrosinase, veratryl alcohol oxidase, lignin peroxidase and azo reductase activities further affirmed the role played by enzymes during degradation of the dye. UV–Visible spectroscopy, Fourier transform infrared spectroscopy (FTIR), high-performance liquid chromatography (HPLC) and gas chromatography–mass spectroscopy (GC–MS) confirmed the biotransformation of dye. A metabolic pathway was proposed based on enzyme activities and metabolites obtained after GC–MS analysis. Therefore, this study affirmed the efficiency of combined biological and AOPs in the treatment of dyes and textile wastewaters in comparison with other methods.

## Introduction

Large volume of untreated dyestuffs and wastewaters released into the environment by textile industries pose a great danger to humans, plants and animals^1^. The threats posed by the indiscriminate disposal of dyes to environmental and health safety is attributed to their aromatic nature^2^. Textile wastewater contains dyes, disinfectants, halogen carriers, solvents, toxic heavy metals, carcinogenic amines, chlorine bleaching, biocides, pentachlorophenol, salts, softeners, surfactants, solvents and free formaldehyde^3^. Most manufacturing and allied industries (like food, leather, textile, and paper) make use of azo dyes in their day-day production of goods and products^4^. The remains of dyes are usually visible in shabbily treated textile wastewaters. This, in turn, causes shallow UV light penetration of ocean beds thus leading to poor water quality, low dissolved oxygen and decline in photosynthetic activities^5^.

Conventional physico-chemicals such as membrane filtration^6^, coagulation^7^, adsorption^8^, and chemical oxidation^9^ have proven to biodegrade the dyes in textile effluent. However, the physical and chemical methods come with attendant disadvantages such as toxic residues formation, membrane fouling, bioaccumulation of sludge and formation of secondary pollutants^10^. To further enhance the reduction of toxic amines in the dyes, conventional biological treatment should be incorporated with advanced oxidation process^11^. Sarria et al.^12^ reported that bioremediation of organic pollutants through AOPs in a single treatment is very arduous, relatively costly and sometimes ineffective.

To overcome the limitation, Advanced Oxidation Process (AOPs) is usually combined with biological treatment in the presence of solar radiation for the biodetoxification of textile azo dyes. This method involves the breakdown of the dye constituents followed by removal of toxic aromatic amines^13^. Bacteria like *A. hydrophila* have proven to be an efficient and promising tool for the removal of textile azo dyes. The use of AOPs enhance biodegradation of textile effluent while bulk reduction of Biological Chemical Demand (BOD) is achieved through biological treatment^14^. Detoxification of dyes is most pronounced of all the biological methods of treatment^15^. Advanced Oxidation Process (AOPs) is usually characterized by the production of radicals (hydroxyl) which are capable of oxidizing aromatic amines in dye wastewater^16^. AOPs are synergistically deployed alongside biological treatment to ensure rapid and efficient textile wastewater treatment^17^. Vilar et al.^18^ suggested the use of sunlight energy systems as Ultraviolet source in the improvement of the oxidation process as an alternative to chemicals. There is paucity of information on the removal of Remazol Yellow RR dye through B-AOPs treatment. Although, different bacteria strains have been deployed in the combined biological and advanced oxidation process (AOPs) treatment of textile dye. However, this is the first report on the degradation of Remazol Yellow RR dye by *A. hydrophila* in combination with AOPs using lesser percentage of hydrogen peroxide.

Hence, this present study is to investigate (1) the potency of biological treatment and advanced oxidation process in the removal of dye (2) determine the enzymes dissipated by *A*. *hydrophila* during biodegradation of Remazol Yellow RR dye (3) propose a biochemical pathway of degradation of the dye. The study revealed that the synergistic biotreatment (with bacteria) and advanced oxidation process (solar radiation) of dye has proven to be cheap, cost-effective and eco-friendly.

## Results

### Comparison of biological, AOPs and combined biological and AOPs

**Figure 1:**
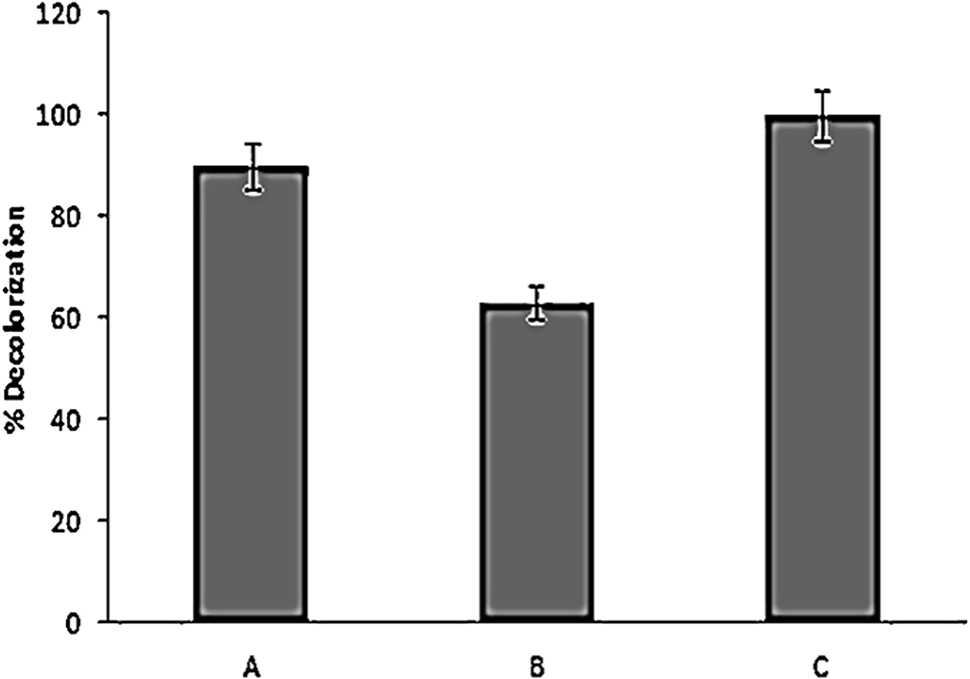
Decolorization percentage of Remazol Yellow RR dye through (**a**) Biological (**b**) AOPs (**c**) Biological + AOPs. Values are plotted as Mean ± Standard Error of Means (*P* ≤ 0.05). Means were separated with Tukey-b. Drawn with GraphPad Prism software version 8.

**Figure 2:**
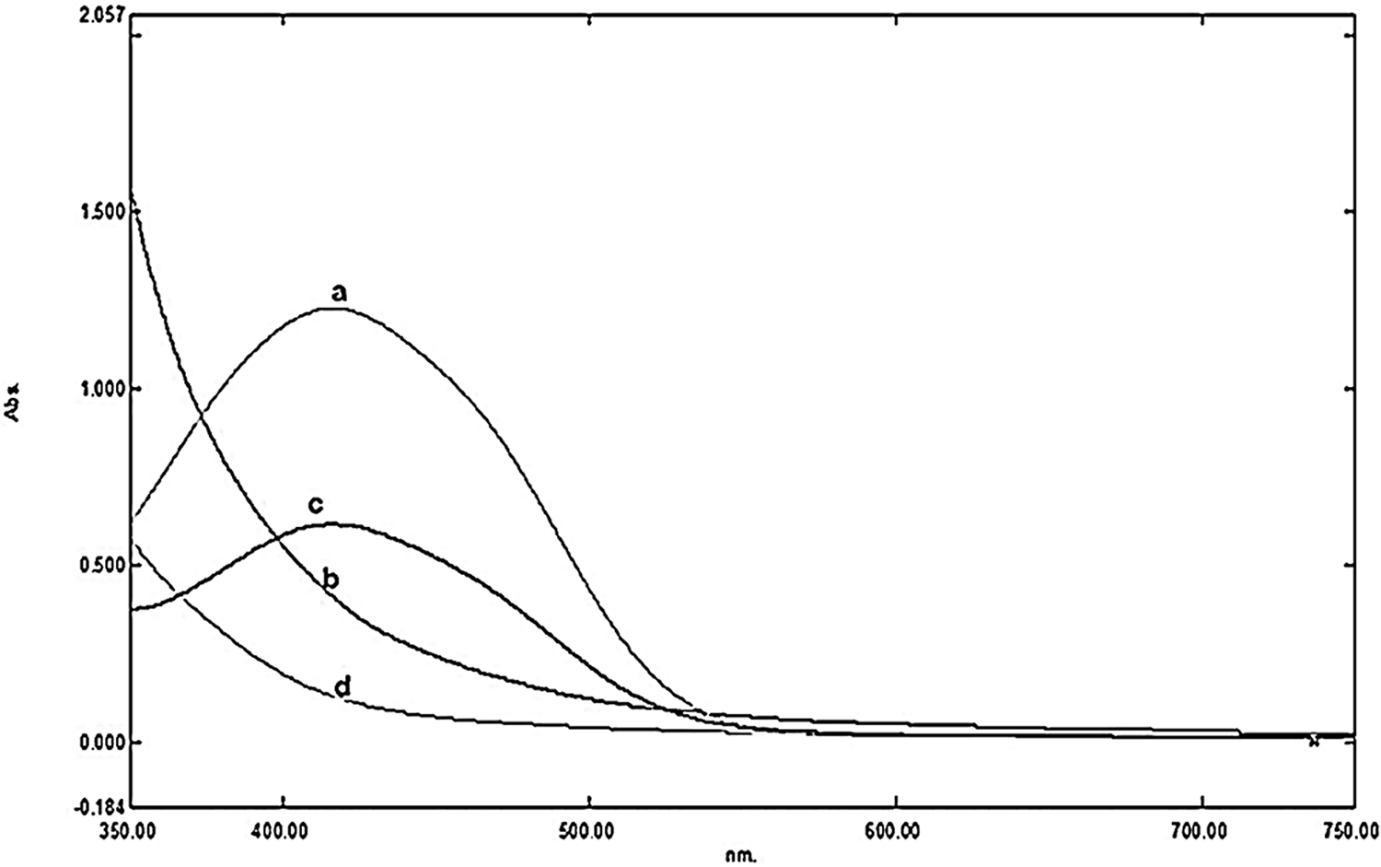
UV–Vis Spectrophotometric analysis of Remazol Yellow RR dye (**a**) Control (**b**) Biological (**c**) AOPs (**d**) Biological + AOPs.

Maximum decolorization (90%) of Remazol Yellow RR was achieved by *A. hydrophila* after 9 h under static condition (Fig. 1a). At present, there is a paucity of information on degradation of dye mediated by desorption and adsorption. On addition of high amount of H_2_O_2_, Advanced Oxidation Process (AOPs) showed less decolorization (63.07%) (Fig. 1b) of decolorization. Degradation becomes quite an arduous task when AOPs are deployed singly because of dye’s complex aromatic structure and nature. The biologically treated sample was centrifuged at 10,000 rpm for 15 min, and subjected to Advanced Oxidation Process (AOPs) with 4% H_2_O_2_ for 3–6 h. The significance values obtained for the treatment with bacteria, AOPs and B-AOPs were 0.823, 0.679 and 0.903 which further affirmed the normality of the experimental data on Remazol Yellow RR dye when subjected to Shapiro–Wilk tests. The skewness and kurtosis conducted to test the distribution of the degradation data further affirmed the normality since they were less than 1 and 2 respectively. The significant value obtained was 0.015 when the degradation data was subjected to Levene statistic. This result was able to ascertain the homogeneous nature of the experimental data. One-way Analysis of Variance (ANOVA) revealed that the percentage degradation data of the dye by bacteria, AOPs and B-AOPs were statistically significant at (*P* ≤ 0.05) when the means were separated with Tukey-b. The treatment showed 100% (Fig. 1c) decolorization within 4 h. After every treatment (biological, AOPs and coupled biological and AOPs) the sample was analyzed by using UV–Vis spectrophotometer in the range of 350–750 nm (Fig. 2).

### Effect of combined treatment (biological, AOPs and B-AOPs) on removal of biological oxygen demand (BOD) and chemical oxygen demand (COD)

**Figure 3:**
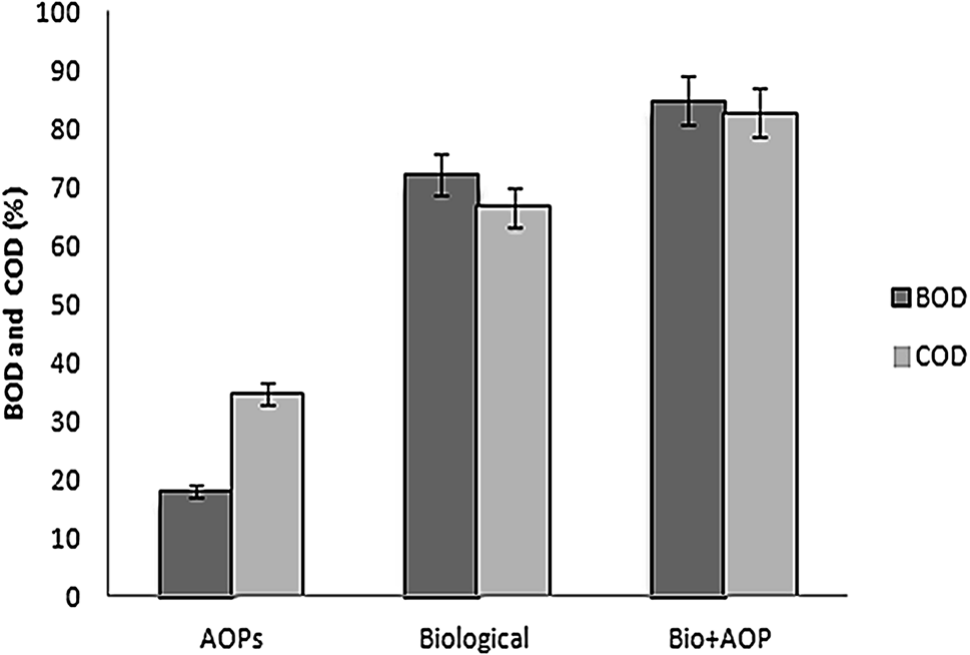
Combined biological and AOPs treatment effects on dye’s BOD and COD. Values plotted as Mean ± Standard Error of Means (*P* ≤ 0.05). Means were separated with Tukey-b. Drawn with GraphPad Prism software version 8.

To ascertain the potency of the combined treatment, all treated samples were tested for reduction in BOD and COD. After completion of AOPs treatment, BOD and COD was reduced by 18 and 34.61% respectively. In biologically treated sample, 72.08 and 66.76% in BOD and COD reduction respectively while 84.88 and 82.76% reduction in BOD and COD was achieved when the treated sample were subjected to combined B-AOPs treatment (Fig. 3). One-way ANOVA revealed that the removal of BOD and COD under synergistic treatment was significantly different (*P* ≤ 0.05) in comparison with individual treatment with bacteria and advanced oxidation process.

### Enzyme activities during decolorization of Remazol Yellow RR dye

Enzyme studies revealed the role played by laccase, tyrosinase, veratryl alcohol oxidase, azo reductase and lignin peroxidase during biodegradation of Remazol Yellow RR. At the end of the decolorization experiment, 194.11, 352.49, 11.53, 288.95 and 198.63 U mL^−1^ min^−1^ of veratryl alcohol oxidase, laccase, tyrosinase and lignin peroxidase was induced. In addition, 148.63 μmol NADH reduced min^−1^ mg protein^−1^ of azo reductase was significantly induced induction after decolorization experiment. The result represents 89.87, 56.50, 47.82, 75.10 and 75.87% inductions in veratryl alcohol oxidase, laccase, tyrosinase and lignin peroxidase and azo reductase activities respectively after complete decolorization of Remazol Yellow RR (Table 1). The skewness and kurtosis of dye degradation by the bacteria, AOPs and B-AOPs were 0.823, 0.679 and 0.903. This suggests that the data were normally distributed since they were less than 1 and 2 respectively. The significant value (0.019) was observed when the data was subjected to Levene statistic which affirmed the homogeneity in the variances in enzymes induction data. One-way ANOVA revealed substantial significant difference (*P* ≤ 0.05) in all the enzyme activity tests before and after treatment of the dye with *A*. *hydrophila* when the means were separated with Tukey-b. Laccase activities was found to be the highest while tyrosinase enzyme was least secreted during the decolorization experiment. Our finding revealed pivotal role played by enzymes in enhancing the decolorization of the dye.

**Table 1:** Enzyme activities in Remazol Yellow RR before and after dye degradation by *A. hydrophila* SK 16.

Enzymes	Before decolorization	After decolorization
Veratryl alcohol oxidase^a^	124.03 ± 0.59	194.11 ± 0.56*
Laccase^a^	185.64 ± 0.01	352.49 ± 1.51*
Tyrosinase^a^	7.80 ± 0.05	11.53 ± 0.09*
Lignin peroxidase^a^	165.02 ± 0.21	288.95 ± 0.16*
Azo reductase^b^	84.51 ± 0.66	148.63 ± 0.34*

Data presented are mean ± standard error of means of three replicate experiments.

*P ≤ 0.05.

^a^Activity in U mL^−1^ min^−1^.

^b^μmol NADH reduced min^−1^ mg protein^−1^.

### Characterization of dye metabolites with Fourier transform infrared spectroscopy (FTIR), high-performance liquid chromatography (HPLC) and gas-chromatography mass spectrometry (GC–MS) analysis

**Figure 4:**
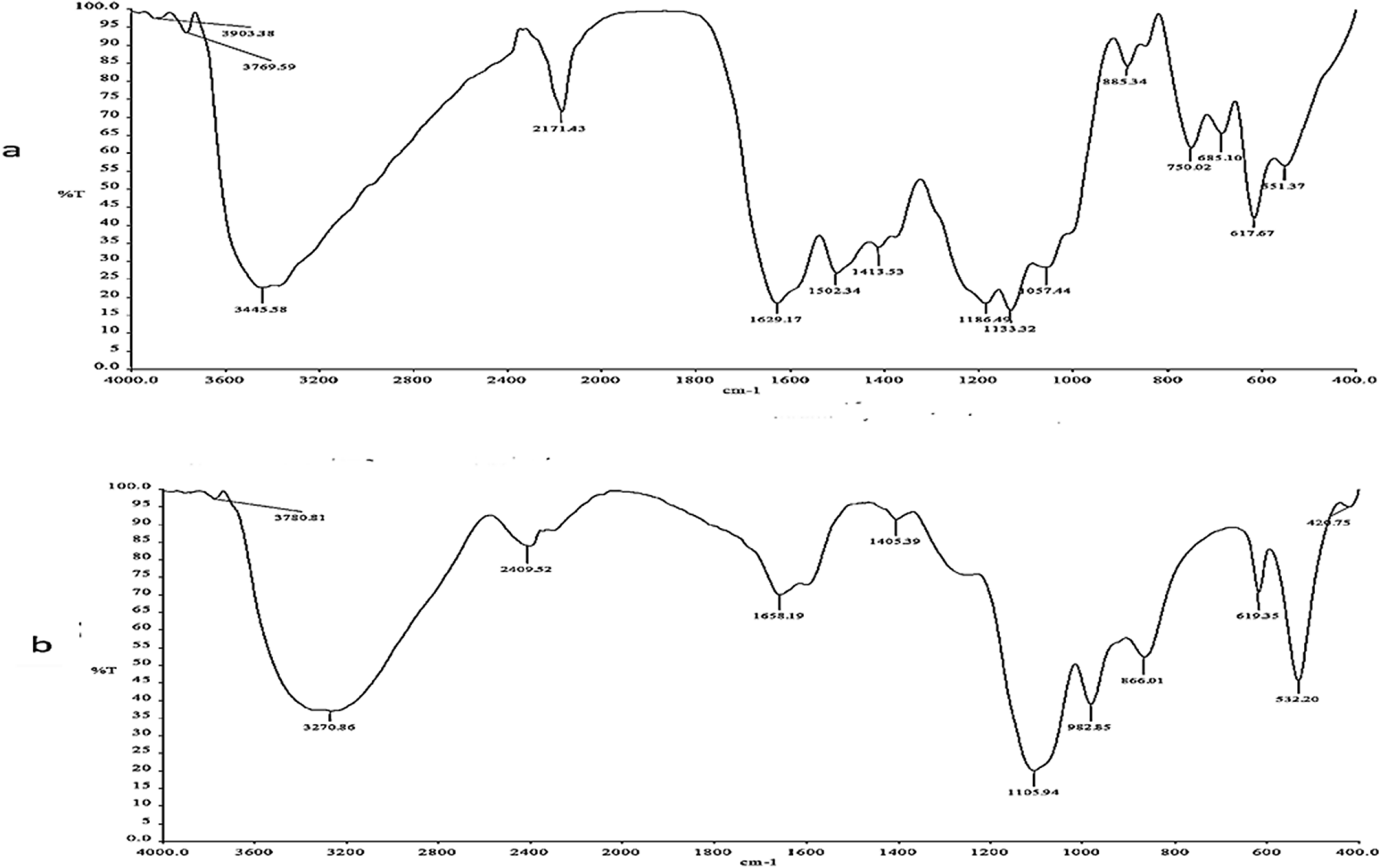
FTIR analysis of Remazol Yellow RR (**a**) control dye (**b**) metabolites.

FTIR spectrum of Remazol Yellow RR showed peaks at 3445, 1629, 1502, 1413, 1186, 1133, 1057, 885, 750, 685, and 617 cm^−1^ which represents to the presence of N–H stretching, N = N (azo bond), N=O stretching, CH_3_ deformation, S=O symmetric stretching sulphonic acid, C–O stretching, C–N stretching, C–H stretching, C–Cl stretching (chlorine group), C–H deformation, C–S stretching sulphur group (Fig. 4a). Products (metabolites) obtained upon biodegradation (total) of Remazol Yellow RR by *A. hydrophila* SK16 showed disappearance of major peaks and formation of several new peaks at 3270, 1658, 1405, 1105, 982, 866, and 619 cm^−1^ which represents to O–H stretching, C = C stretching, N=O bend, C–OH stretching, CH_2_ stretching, C-H stretching and C-H bend (Fig. 4b).

**Figure 5:**
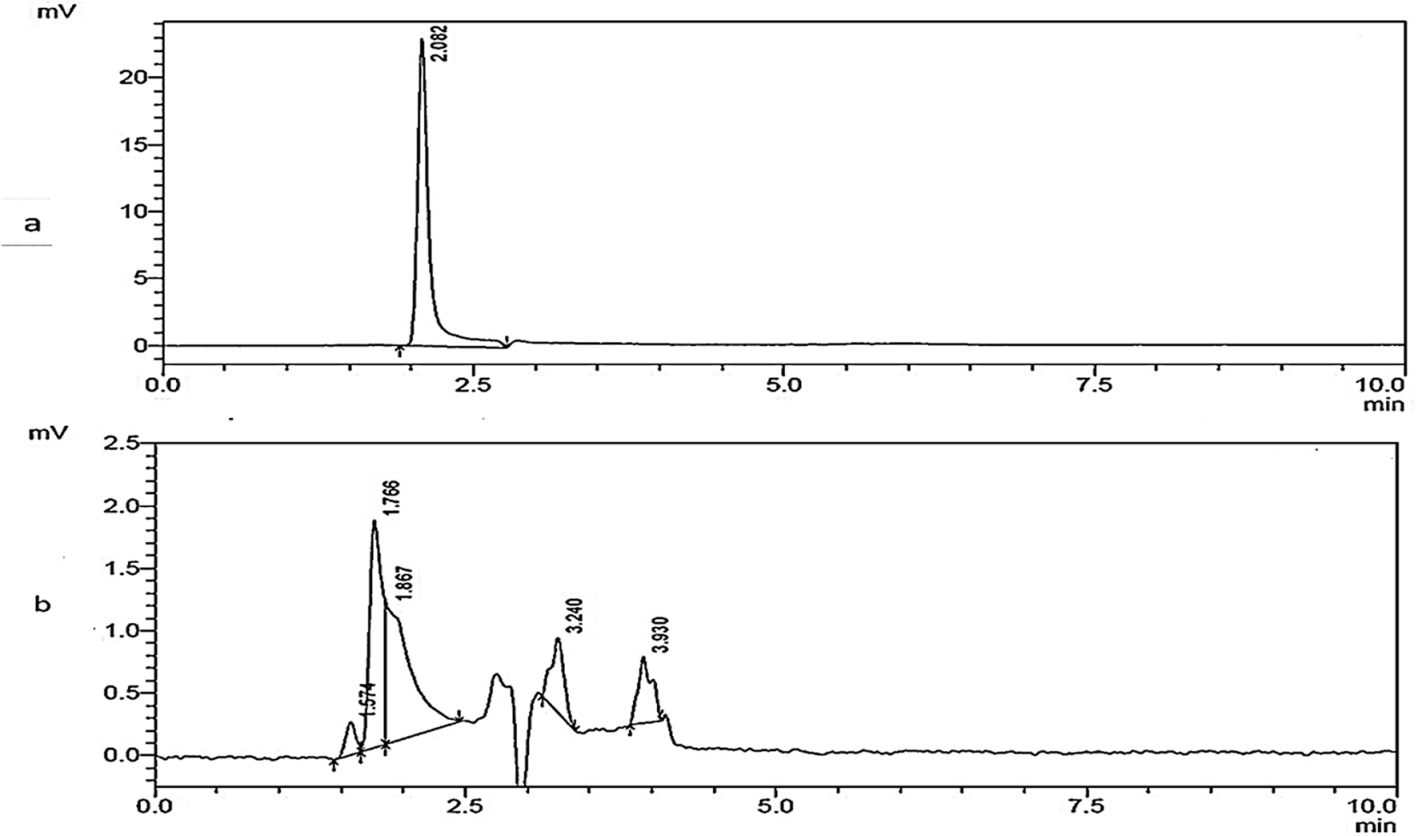
HPLC spectra peaks of Remazol Yellow RR dye (**a**) control sample (**b**) metabolite.

HPLC analysis further confirmed appearance of various metabolites from control dye. Control sample showed a major peak at 2.029 min retention time (Fig. 5a), whereas biodegraded sample showed 5 new peaks with retentions times (1.574, 1.766, 1.867, 3.240 and 3.930 min respectively) (Fig. 5b). The HPLC analysis confirmed the single major peak in the control Remazol Yellow RR dye has been biodegraded into different peaks (five-5) evidently shown by the times (retention). This analysis further affirmed the degradation of the dye.

**Figure 6:**
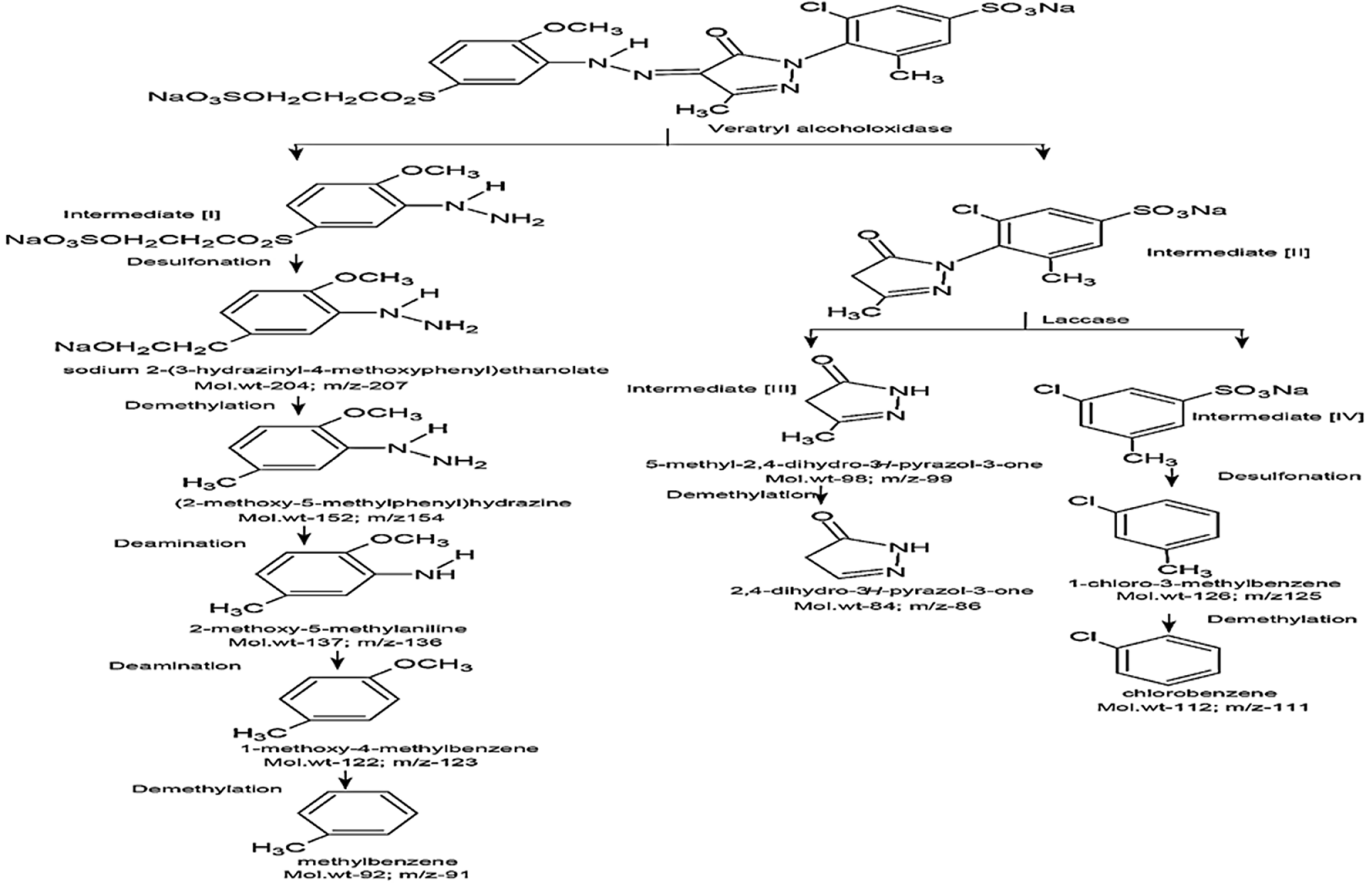
Metabolic pathways for Remazol Yellow RR biodegradation by *A. hydrophila* SK16. Drawn with ChemSketch software version 11.02.

Remazol Yellow RR dye metabolites after biodegradation by *A. hydrophila* SK16 was analyzed using Gas Chromatography and Mass Spectroscopy (GCMS) elucidated with peaks in mass spectra (data not shown). Primarily, veratryl alcohol oxidase carried symmetric cleavage of Remazol Yellow RR which yielded Intermediate [I] and Intermediate [II]. Desulfonation of Intermediate [I] gave rise to sodium 2-(3-hydrazinyl-4-methoxyphenyl) ethanolate with the retention time (16.08 min) and peak (mw = 204; m/z = 207), followed demethylation resulted in the formation of 2-methoxy-5-methylphenyl)hydrazine with a retention time (22.20 min) and peak (mw = 152; m/z = 154). Further, deamination provided 2-methoxy-5-methylaniline with the retention time of (17.81 min) and peak (mw = 137; m/z = 136), followed deamination resulted in the formation of 1-methoxy-4-methylbenzene with a retention time (21.09 min) and peak (mw = 122; m/z = 123), followed demethylation resulted in the formation of methylbenzene with a retention time (13.97 min) and peak (mw = 92; m/z = 91). Symmetric cleavage of Intermediate [II] by laccase resulted in the formation of Intermediate [III] identified as 5-methyl-2,4-dihydro-3*H*-pyrazol-3-one with retention time (14.39 min) and peak (mw = 98; m/z = 99) and Intermediate [IV]. Further, demethylation of Intermediate [III] resulted in the formation of 2,4-dihydro-3*H*-pyrazol-3-one with a retention time of 10.37 and peak (mw = 84; m/z = 86). Desulfonation of intermediate [IV] provided 1-chloro-3-methylbenzene with the retention time (15.14 min) and peak (mw = 126; m/z = 125), followed demethylation resulted in the formation of chlorobenzene with the retention time (13.26 min) and a mass peak (mw = 112; m/z = 111). A possible metabolic pathway was proposed for Remazol Yellow RR by *A. hydrophila* SK16 (Fig. 6).

## Discussion

The UV–Vis spectral results revealed that synergistic treatment with *A. hydrophila* and AOP accounted for the total disappearance of the major peak in Remazol Yellow RR dye (control) than individual treatment with the bacteria and AOP respectively. Kalme et al.^19^ implied in an earlier work that low decolorization efficiency in aerobic state is due to the interplay of molecular forces between oxygen and azo compounds. Similar results were reported by Kalyani et al.^20^ when *Pseudomonas* sp. SUK1 was deployed in dye degradation. The power of microbial cells to adsorb dye accounted for decolorization efficiency over a period^20^. Albeit, biodegradation via oxidation (chemical) is very expensive due to the oxidation intermediates produced during treatment. Furthermore, the intermediates are more resistant to complete chemical oxidation and furthermore consume energy relative to treatment time^21^. Harrelkas et al.^22^ reported that it is highly efficient if biological treatment is incorporated to OAPs in order to enhance overall treatment efficiency. First biological treatment decreases concentration of compounds that may compete for chemical oxidation, thus increasing efficiency and lowering treatment cost^23^. In non-biodegradable textile effluent, the coupled treatment not only achieves efficient decolorization, but also significantly reduces BOD, COD and TDS^24^. The findings in this study revealed higher decolorization potency than the efficiencies recorded by Tantak and Chaudhari^25^ when Reactive Blue 13 and Reactive Blue 5 was subjected to combined B-AOPs treatment. However, this study corroborated the findings of Lodha and Chaudhari^26^ who reported 99% removal of color when different dye solutions were subjected to combined AOPs and biological treatment. Furthermore, this study was in agreement with the reports of Alvares et al.^27^ on the efficiency of combined B-AOPs in the removal of colors in textile wastewaters. The desulfonation, demethylation and asymmetric cleavage which led to the production of metabolites of Remazol Yellow RR dye as depicted in the metabolic pathway (proposed) was facilitated by high induction of laccase during degradation. The reports on biodegradation of Reactive Red 2 by Kalyani et al^20^ further corroborated the pivotal role played by laccase. Furthermore, these results were in agreement with previous reports of Parshetti et al^28^ who revealed the crucial role played by NADCH-DCIP reductase and laccase in the removal of malachite green by *Kocuria rosea*. Evidently, the spectra analysis (FT-IR) of the metabolites showed disappearance and proliferation of new peaks which are different from the peaks observed in the control (Remazol Yellow RR) dye. The structural changes in the fingerprint and functional group regions of the Remazol Yellow RR dye spectra strongly suggests biodegradation. In addition, formation of new peaks formed at different retention times in the treated Remazol Yellow RR dye HPLC spectrum equally affirmed the breakdown of dye. These results were corroborated the reports of Jadhav et al^5^_._

## Methods

### Dyes and chemicals

Remazol Yellow RR dye was obtained from Tamil Nadu, India (Jamara textile industry). Veratryl alcohol, methyl red, L-ascorbic acid, Catechol and nutrient broth were obtained from Pvt Laboratories (Hi Media), Mumbai. Hydrogen peroxide was procured from Merck Mumbai. Other reagents used were of high purity and analytical grade.

### Culture maintenance and decolorization

*Aeromonas hydrophila* SK16 used in the present work was isolated previously in our laboratory from textile contaminated soil^29^. The culture was maintained at 4˚C on nutrient agar slant (Nutrient Agar: NaCl-5 g, Beef extract-1.5 g, Yeast extract-1.5 g, and Agar-15 g). Decolorization study was carried out in Erlenmeyer flask (250 mL) containing nutrient broth of aforementioned composition. The dye (100 mg L^−1^) was added to a pre-grown culture of *A. hydrophila* and incubated at 37˚C under static condition. The pH was adjusted to 8 in pre-grown culture with the addition of NaOH. A 5 mL aliquot was withdrawn at regular intervals, centrifuged at 10,000 rpm for 15 min and decolorization was monitored with UV–Vis Spectrophotometer (Shimadzu 1800) at wavelength (λ_max_ = 560 nm). The control experiments were made of flasks with no bacteria cells. All the experiments were performed in triplicates. Decolorization percentage was calculated using the formula:

1 $$ Decolorization \left( \% \right) = \frac{Initial \,absorbance - Final \,absorbance}{{Initial\, absorbance}} \times 100 $$

### Experiments on advanced oxidation process (AOPs)

The AOPs was carried out in Erlenmeyer flask (250 mL) containing Remazol Yellow RR dye (100 mg L^−1^) with 4% hydrogen peroxide. The percentage of H_2_O_2_ used for this study was based on the initial screening done with variations from 1 to 10%. The setup was placed for 6 h under UV radiation. This procedure was repeated in dark conditions. The control experiment was made of flasks with no H_2_O_2_. The light intensity was monitored with Lux meter (Lutron LX-101, Taiwan).

### Biological and advanced oxidation process (AOP) treatment

The biodegraded sample was centrifuged at 10,000 rpm for 15 min. The supernatant was subjected to AOPs with 4% H_2_O_2_ for 3–6 h. Lux meter (Lutron LX-101, Taiwan) was used to measure Light intensity. The sample was thereafter analyzed with UV–Vis spectrophotometer (Shimadzu 1800, Japan).

### Characterization of Remazol Yellow RR

Biological Oxidation Demand (BOD) and Chemical Oxidation Demand (COD) of Remazol Yellow RR were characterized before and after dye degradation^30^.

### Cell-free extract preparation for enzyme assays

Bacterial cells were cultured in nutrient broth for 24 h and centrifuged at 10,000 rpm for 15 min. The supernatant was removed and the bacterial cells (75 mg L^−1^) were suspended in potassium phosphate buffer (50 mM, pH 7.4), homogenized in a glass homogenizer and sonicated (sonics-vibracell) at 40 amplitude at 4˚C and giving 8 strokes, each of 40 s with 2 min interval. This sample was used for enzyme studies without further centrifugation. Control experimental set up contains no dye.

### Enzyme assays

Activities of laccase and veratryl alcohol oxidase were assayed spectrophotometrically by using UV–Vis spectrophotometer (Shimadzu 1800, Japan). The total volume of 2 mL was contained 1 mM veratryl alcohol, citrate buffer (pH 3)—0.1 M and 0.2 mL enzyme source. This was done to determine veratryl alcohol oxidase. Oxidation of veratryl alcohol was monitored by increasing of absorbance at 310 nm due to formation of propanaldehyde^31^. Laccase activity was determined in a 2 mL of reaction mixture containing 10% of ABTS in 20 mM potassium phosphate buffer (pH 4) and the increased optical density was measured at 420 nm^32^. Lignin peroxidase assay was performed in a total 2.5 mL volume comprising tartaric acid (250 mM) and n-propanol (100 mM). The propanaldehyde production was estimated at 300 nm as reported by Kalyani et al.^20^. Tyrosinase activity was calculated in a reaction mixture of 2 mL, containing in 0.1 M phosphate buffer (pH 7.4) with 0.01% catechol at 495 nm^24^. Azoreductase assay was carried out using Methyl red as substrate as reported by Kurade et al.^33^.

### Extraction and analysis of metabolites produced during biodegradation

After complete biodegradation, the set up was centrifuged (at 10,000 rpm for 15 min). Ethyl acetate was proportionately mixed with the supernatant in order to extract the metabolites produced during biodegradation. Rotary evaporator was used to dry the extracts over anhydrous Na_2_SO_4_. The dried sample was dissolved in HPLC grade methanol and used for analytical studies. The changes of surface functional groups of Remazol Yellow RR before and after biodegradation was investigated by using FTIR Perkin Elmer (RX I)^34^. HPLC analysis was conducted using Jadhav et al.^5^ method. The Shimadzu LC 40,102,010 instrument was used for HPLC study connected with C18 column. The solvent methanol was used in mobile phase with 1 mL min^−1^ flow rate and analysis was done at 470 nm^23^.

Gas Chromatography (GC)—45XGC-44 coupled with Scion MS-40 Mass Spectroscopy (Bruker) was used in analyzing metabolites obtained after biotransformation. Voltage of (70 eV) was used while the carrier gas was made of helium with 1 mL min^−1^ (flow rate) and 26 min (duration). DB-WAX column (0.25 mm-30 mm) was used for the GC analysis initially set at the operating temperature mode. The column (oven) temperature was increased steadily by 10 °C per minute to 250 °C. The operating condition was kept for 26 min. from the initially programmed temperature of 80˚ C. ChemSketch software version 11.02 was deployed in sketching the metabolic products, hence the pathway (proposed) while the metabolites were identified based on comparison with mass spectra available in the NIST database.

### Statistical analysis

All experiments were performed in triplicates. Data and graphs were presented as Mean ± Standard Error of Means at 95% Confidence Interval. Normality and homogeneity of variances of the data on Remazol Yellow dye degradation and enzymes induction were conducted with Shapiro–Wilk and Levene statistic tests respectively^35^. One-way Analyses of Variance (ANOVA) was carried out to affirm the homogenous nature of the data on degradation of dye and enzyme induction. Statistical analysis (ANOVA) (*P* ≤ 0.05) and graphs was performed with GraphPad Prism software version 8^35^.

## Conclusion

The synergistic effect of Advanced Oxidation Process (AOPs) and biological treatment developed by incorporating *A. hydrophila* SK16 and 4% H_2_O_2_ for Remazol Yellow RR dye biodegradation was reported. This combined treatment achieved 100% decolorization, 84.88% BOD and 82.76% COD reduction. The biodegradation process elucidated substantial induction of laccase and veratryl alcohol oxidase. UV–vis spectroscopy, FTIR, HPLC and GC–MS analysis proved that degradation of Remazol Yellow RR by *A. hydrophila* SK16. Therefore, the combined B-AOPs provides cost effective, non-energy demanding and ecofriendly treatment for managing textile dye pollution in the environment.

## Data Availability

All experimental data would be made freely available and accessible upon request by the corresponding author.
